# Third-person self-talk facilitates emotion regulation without engaging cognitive control: Converging evidence from ERP and fMRI

**DOI:** 10.1038/s41598-017-04047-3

**Published:** 2017-07-03

**Authors:** Jason S. Moser, Adrienne Dougherty, Whitney I. Mattson, Benjamin Katz, Tim P. Moran, Darwin Guevarra, Holly Shablack, Ozlem Ayduk, John Jonides, Marc G. Berman, Ethan Kross

**Affiliations:** 1 Department of Psychology, Michigan State University, East Lansing, Michigan USA; 2 Department of Psychology, University of Michigan, Ann Arbor, Michigan USA; 3 Department of Psychology, University of California, Berkeley, California, USA; 4 Department of Psychology, University of Chicago, Chicago, Illinois USA; 5 School of Medicine, Emory University, Atlanta, Georgia

## Abstract

Does silently talking to yourself in the third-person constitute a relatively effortless form of self control? We hypothesized that it does under the premise that third-person self-talk leads people to think about the self similar to how they think about others, which provides them with the psychological distance needed to facilitate self control. We tested this prediction by asking participants to reflect on feelings elicited by viewing aversive images (Study 1) and recalling negative autobiographical memories (Study 2) using either “I” or their name while measuring neural activity via ERPs (Study 1) and fMRI (Study 2). Study 1 demonstrated that third-person self-talk reduced an ERP marker of self-referential emotional reactivity (i.e., late positive potential) within the first second of viewing aversive images without enhancing an ERP marker of cognitive control (i.e., stimulus preceding negativity). Conceptually replicating these results, Study 2 demonstrated that third-person self-talk was linked with reduced levels of activation in an a priori defined fMRI marker of self-referential processing (i.e., medial prefrontal cortex) when participants reflected on negative memories without eliciting increased levels of activity in a priori defined fMRI markers of cognitive control. Together, these results suggest that third-person self-talk may constitute a relatively effortless form of self-control.

## Introduction

We all have an internal monologue that we engage in from time to time; an inner voice that guides our moment-to-moment reflections^1–3^. Although people frequently engage in such “self-talk”, recent findings indicate that the language they use to refer to the self when they engage in this process influences self-control. Specifically, using one’s own name to refer to the self during introspection, rather than the first-person pronoun “I”, increases peoples’ ability to control their thoughts, feelings, and behavior under stress^4–6^.

But just how easy is it for people to control their emotions via third-person self-talk ? Emotion regulation, as with many forms of self-control, is typically thought of as an effortful process e.g.^7^, that depends heavily on cognitive control mechanisms to muffle emotional responses^8–10^. Might third-person self-talk constitute a relatively effortless form of emotional control that does not require additional cognitive control processes above and beyond those recruited when people typically reflect on negative experiences? Here we suggest that it does.

This prediction is motivated by the observation that people almost exclusively use names to refer to other people. Thus, there is a tight coupling between using proper names, and thinking about others—a coupling that is so tight that we expected using one’s own name to refer to the self would virtually automatically lead people to think about the self similarly to how they think about someone else. If this prediction is correct, and if it is indeed easier for people to reason calmly about other people’s emotions than their own^4, 5^, then third-person self-talk should be linked with reductions in emotional reactivity but not enhancements in cognitive control.

We tested these predictions by asking participants to reflect on their feelings associated with viewing aversive images from the International Affective Picture System (Study 1) and recalling painful autobiographical memories (Study 2) using either **“I”** or **their name** while measuring neural activity via event-related brain potentials (ERPs; Study 1) and functional magnetic resonance imaging (fMRI; Study 2).

**Figure 1:**
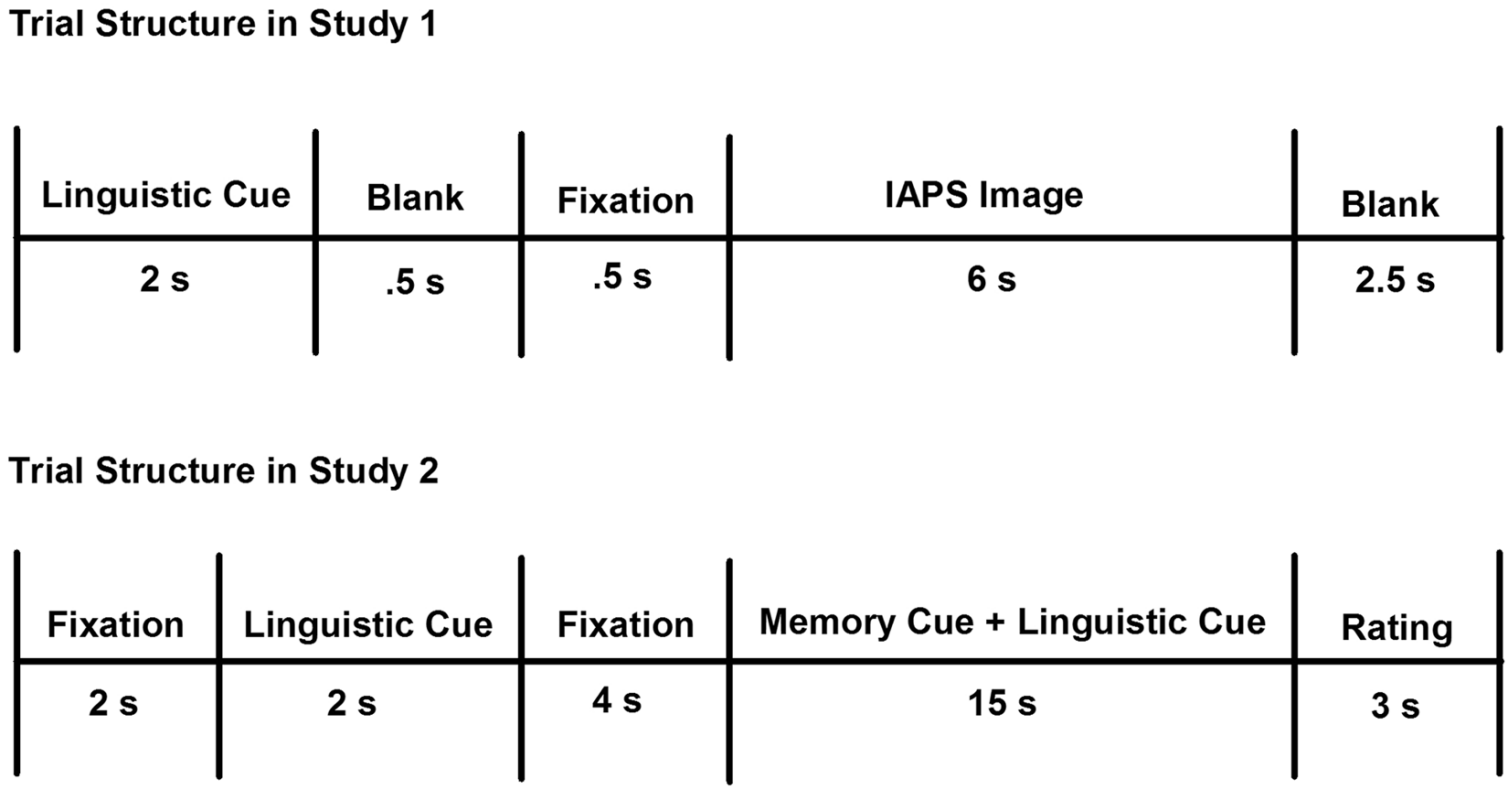
Visual depiction of trial structures in Study 1 (top) & Study 2 (bottom). In Study 1, participants first viewed a linguistic cue (“First-Person” or “Third-Person”) for 2 s that directed them to either use I or their own name when thinking about the following picture. Next, a blank screen was presented for 500 ms followed by a white fixation cross lasting 500 ms. Following the fixation cross, an IAPS image was displayed for 6 s. A blank screen then appeared for 2.5 s. In Study 2, each trial began with a 2 s fixation cross. Subsequently, participants saw a linguistic cue for 2 s (“I” or their own name). Next, they saw another fixation cross for 4 s. A memory cue-phrase then appeared on the center of the screen for 15 s, signaling participants to reflect on an autobiographical experience they had generated previously. The same linguistic cue that participants saw at the beginning of the trial appeared beneath the memory cue to ensure that participants continued to reflect on the memory using the appropriate part of speech. Finally, participants had 3 s to rate how they felt using a five-point scale (1 = not at all negative; 5 = very negative).

In Study 1, we measured ERPs while participants viewed standardized images depicting arousing negative and neutral scenes under two conditions. In the First-Person condition, participants asked themselves *“…what am* [*I*] *feeling right now? ”*; in the Third-Person condition, they asked themselves, “*…what is* [*Participants*’ *Name*] *feeling right now*” (Fig. 1, top panel). ERPs have been extensively used to identify the neural mechanisms supporting people’s ability to control emotional responses. This work consistently reveals two waveforms that are involved in emotion regulation: the late positive potential (LPP) and the stimulus preceding negativity (SPN). The LPP is a robust marker of emotional reactivity^9, 11^. It is enlarged to negative and positive stimuli relative to neutral stimuli, especially when such stimuli are self-relevant^12^, and is closely coupled with subjective ratings and physiological markers of arousal^11, 13^. The frontally distributed SPN, on the other hand, indexes cognitive control processes^9, 14^. Importantly, an extensive body of research indicates that the LPP is attenuated and the SPN amplified during the implementation of effortful emotion regulation strategies such as cognitive reappraisal^15–17^. Thus, our analyses focused on these waveforms. Based on our theoretical framework, we predicted that third-person self-talk would lead to reductions of the LPP elicited by aversive images but no change in the SPN.

In Study 2, we extended our ERP study in two ways. First, while the standardized images used in Study 1 are useful for studying emotion under tightly controlled conditions, many of the situations that require self-control in daily life are elicited by thinking about idiosyncratic negative experiences. Thus, in Study 2 we used an autobiographical memory paradigm to elicit negative emotion to examine how third-person self-talk operates in a more ecologically valid context (see Fig. 1, bottom panel).

Second, whereas the ERPs used in Study 1 provide information about the temporal dimensions of self-referential emotional processing and cognitive control, they do not provide detailed information about the specific brain structures involved. Therefore, in Study 2 we used fMRI to test whether third-person self-talk would reduce activations in a priori identified brain regions that are commonly implicated in thinking about the self versus others (e.g., medial prefrontal cortex;^18^) and emotional reactivity (i.e., the amygdala;^8^) *without* increasing activation in fronto-parietal regions that support cognitive control^8^.

Using both of these neuroimaging methods allowed us to pursue converging evidence for our hypothesis across the temporal and spatial dimensions of self-referential emotional processing and cognitive control. Employing two different emotion elicitation paradigms further allowed us to evaluate whether our hypothesized effects of third-person self-talk would generalize across relatively more standardized versus ideographic stimuli. Together, these two experiments provide a multi-method test of our hypothesis regarding third-person self-talk as a relatively effortless form of self-control.

## Results

### Study 1

As a preliminary manipulation check, we examined participant compliance with the use of the first- vs. third-person pronouns when focusing on their feelings using a 1 (not at all) to 7 (all the time) likert scale (see Method & Materials section below for more detail). Overall, compliance with the instructions was superb. The mean ratings for the first- (*M* = 6.66; *SD* = .86) and third- (*M* = 6.28; *SD* = 1.03) person conditions were both well above the scale midpoint (*ts*(28) > 11.88, *ps* < 0.001, *ds* > 3.13) and not significantly different from each other (*t*(28) = 1.78, *p* > 0.05, *d* = 0.40), demonstrating that participants could easily implement both sets of instructions.

**Figure 2:**
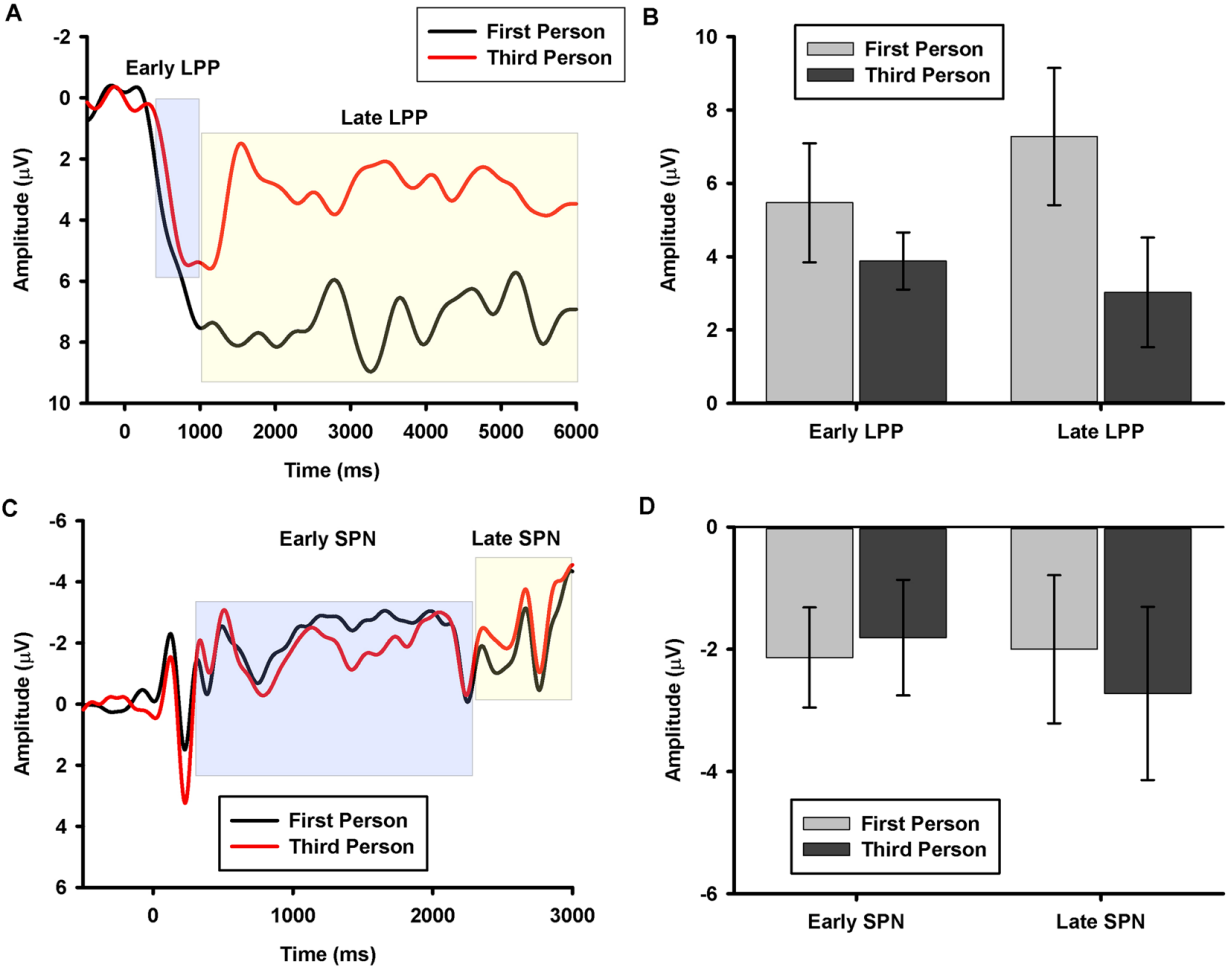
Study 1 (ERP) Results. **(A)** Picture-locked ERP waveforms at representative left posterior superior site depicting the larger late, but not early, negative-neutral LPP difference wave in the First-Person compared to Third-Person condition in Study 1. Picture onset occurs at 0 ms. Blue shaded area represents time window for analysis of early LPP; yellow shaded area represents time window for analysis of the late LPP. **(B)** Bar graph depicting the larger negative-neutral difference score in the First-Person compared to the Third-Person for the late, but not early, LPP. Error bars reflect+/− 1 *SEM*. **(C)** Cue-locked ERP waveforms at frontocentral recording sites depicting the null main effect of type of self-talk, averaged across valence, on the SPN in Study 1. Cue onset occurs at 0 ms and picture onset occurs at 3000 ms. Blue shaded area represents time window for analysis of early SPN; yellow shaded area represents time window for analysis of the late SPN. **(D)** Bar graph depicting the null main effect of type of self-talk, averaged across valence, on the early and late SPN. Error bars reflect+/− 1 *SEM*.

We first examined the effect of third-person self-talk on the LPP. Because the main aim of the current investigation was to determine the emotion regulatory effects of third-person self-talk, the focus of our LPP analysis was on the interaction between Valence (negative vs. neutral images) and Self-Talk Strategy (first-person vs. third-person self-talk). Specifically, we tested whether third-person self-talk reduced the emotional modulation (i.e., negative vs. neutral) of the LPP by performing separate repeated measures analyses of variance on the early (400 ms – 1 s) and late (1–6 s) time windows (for similar approach, see^9, 19^). Figure 2A displays the stimulus-locked ERP waveforms and Fig. 2B displays the mean amplitudes for the emotional modulation (negative minus neutral difference depicted for both A and B) of the early and late LPP in the first- and third-person self-talk conditions.

Analysis of the early time window revealed no interaction between Valence and Self-Talk Strategy (*F*_*1,28*_ < 1, *p* = .36, *η* ^*2*^_*p*_ = 0.03). Figure 2B (left side) shows that the negative minus neutral difference scores were identical for first- and third-person trials. However, a significant interaction between Valence and Self-Talk Strategy emerged during the late time window (*F*_1,28_ = 5.18, *p* = .03, *η* ^*2*^ *s*_*p*_ = 0.16). As predicted, on first-person trials, the LPP was significantly greater for negative than neutral images (*t*(28) = 3.07, *p* < 0.01, *d* = 0.89; Fig. 2B right side, light grey bar shows significant difference between negative and neutral images). However, on third-person trials the LPP for negative and neutral images were indistinguishable (*t*(28) = 1.50, *p* = 0.15, *d* = 0.23; Fig. 2B right side, dark grey bar shows non-significant difference between negative and neutral images). Direct comparison of the negative minus neutral difference scores for the first-person versus third-person conditions demonstrated the predicted larger difference score for the first- compared to third- person condition (*t*(28) = 2.18, *p* = 0.03, *d* = 0.82; Fig. 2A and B; see also Fig. S2 for topographical distribution of this difference effect).

Next, we examined whether third-person self-talk modulated the SPN. Because we aimed to test whether third-person self-talk cues engaged greater cognitive control than first-person self-talk cues, the focus of our SPN analysis was on the main effect of Self-Talk Strategy (first-person vs. third-person self-talk) – the interaction between Self-Talk Strategy and Valence was irrelevant for the SPN given that participants were unaware of the valence of the upcoming picture at the time of the presentation of the Self-Talk Strategy cue. Specifically, we tested whether third-person self-talk enhanced the amplitude of the SPN by performing separate repeated measures analyses of variance on the early (300–2300 ms) and late (2300 ms – 3 s) time windows. Figure 2C displays the stimulus-locked ERP waveforms and Fig. 2D displays the mean amplitudes for the averaged main effect of Self-Talk Strategy, averaged across valence, on the early and late SPN. Consistent with the hypothesis that third-person self-talk does not recruit cognitive control processes, the main effect of Self-Talk Strategy on the early (*F* = .10, *p* = 0.76) and late (*F* = 0.31, *p* = 0.58) SPN was non-significant (Fig. 2C and D; see also Fig. S3 for topographical distribution of this null effect). Moreover, as expected, there were no interactions between Self-Talk Strategy and Valence for either SPN time window (*F*s < 1, *p*s > 0.54).

### Study 2

We contrasted the hemodynamic response on trials in which participants recalled negative autobiographical experiences and then analyzed their feelings surrounding the events using ***I*** or ***their own name***. Participants rated how they felt after each trial using a five-point scale, with lower numbers reflecting less distress.

As predicted, participants reported experiencing more negative affect on I trials (*M* = 3.82, *SD* = 0.55) than Name trials (*M* = 3.24, *SD* = 0.60; *t*(49) = 7.03, *p* < 0.001, *d* = 1.01). Next we examined whether third-person self-talk (compared to first-person self-talk) led to relatively less activation in brain regions that support thinking about the self versus others and emotional reactivity. Because our hypotheses were theory driven, we performed region of interest (ROI) analyses on (a) a set of cortical midline regions commonly implicated in thinking about the self versus others (e.g., medial prefrontal cortex/anterior cingulate cortex) and (b) brain regions involved in emotional reactivity (i.e., the amygdala). Both sets of ROIs were identified from recent meta-analyses^18, 20^.

**Figure 3:**
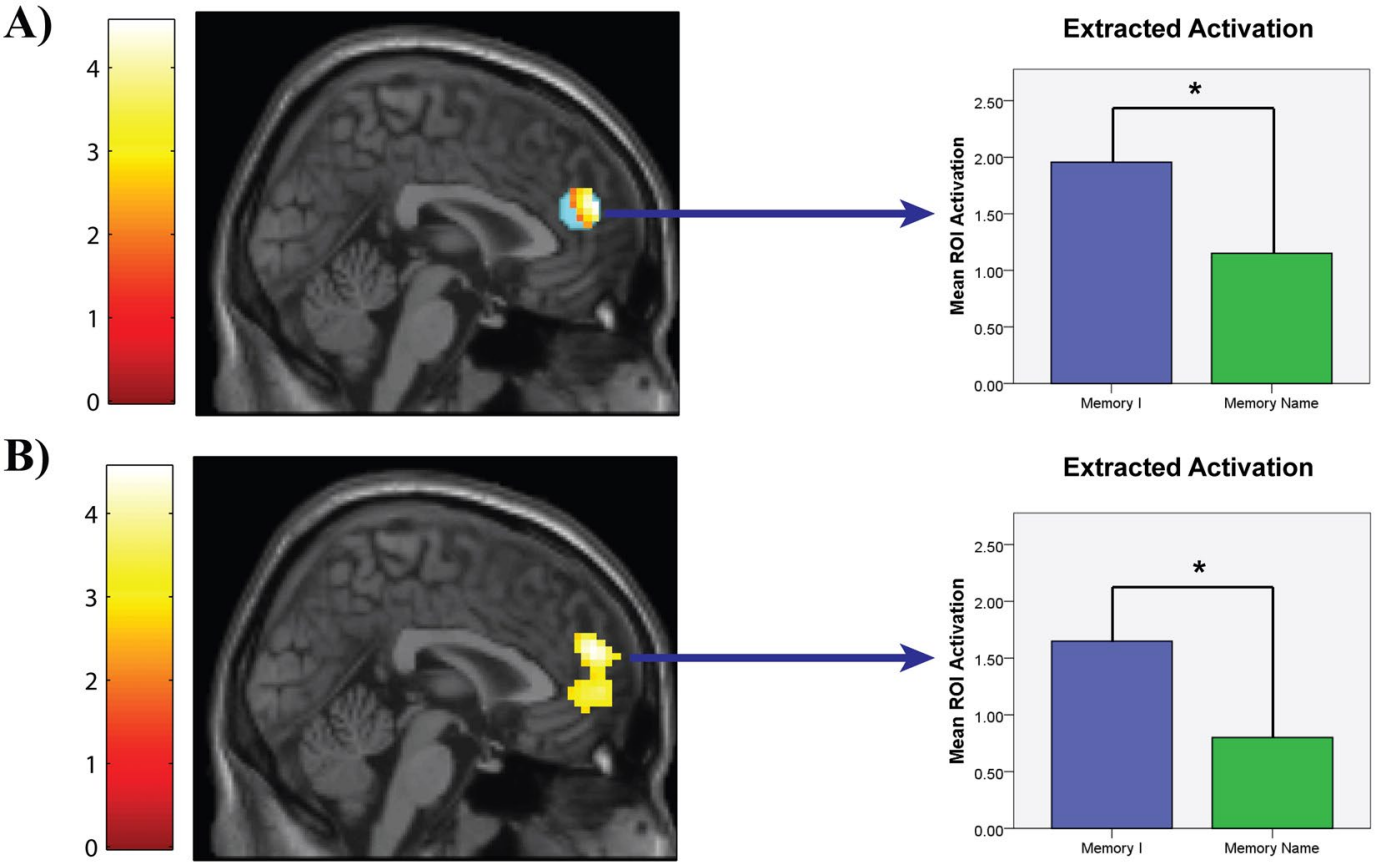
Study 2 (fMRI) Results. Brain imaging results from Study 2 showing that reflecting on negative experiences using “I” instead of one’s own name (I > Name Contrast) is associated with significantly more activity in a region of the medial prefrontal cortex identified as playing a role in self-referential processing in (**A**) a region of interest (ROI colored in blue; activations observed within a priori ROI are overlaid in yellow-orange coloring) and (**B**) whole-brain analysis. Adjacent to each brain map is a bar graph illustrating the extracted mean activation between “I” and “Name” trials from each analysis. * represents *p* < .05.

As Fig. 3A illustrates, third-person self-talk led to significantly less activity in the left medial prefrontal cortex/anterior cingulate cortex ROI, *t* = 4.54, *k* = 143, *p* < .0001, Sphere at MNI *xyz* = −6, 46, 20. Note that this peak activity was still significant after correcting for multiple comparisons (5 ROIs examined; p. 005/5 comparisons = p. 001). No other ROIs had activation above signal and cluster thresholds. Whole-brain analyses were consistent with these results (see Fig. 3B), *t* = 4.54, *k* = 258, *p* < .001, Peak at MNI *xyz* = 2, 53, 22. [Inspection of the distribution of beta values extracted from the I > Name contrast revealed two extreme values – i.e., participants with beta values that were two standard deviations above the sample mean. Excluding these participants from our analyses did not substantively alter any of the results we report here.]

Finally, we examined whether third-person self-talk led to increases in brain regions that support the cognitive control of emotion. Conceptually replicating the Study 1 results, an ROI analysis performed on the fronto-parietal network commonly implicated in the cognitive control of emotion^20^ did not reveal any significant differences between the two conditions. This was true even when the statistical threshold was dropped to p < .01, unconditional, suggesting that failure to observe activations in these ROIs was not a function of statistical power.

## Discussion

Across two neuroscience modalities and two different emotion elicitation procedures, the current findings suggest that third-person self-talk facilitates emotional control without recruiting cognitive control. These results stand in contrast to much of the extant work on self-control, and emotional regulation in particular, which typically conceive of these processes as effortful^8–10, 15^. This is not to say that other forms of automatic self-control do not exist^21, 22^. Rather, our findings add to this work by demonstrating how a linguistic shift that promotes psychological distance from the self modulates emotional responses.

If fronto-parietal mediated cognitive control processes do not enable third-person self-talk, then what does? The theoretical framework and behavioral results^4^ driving this work contend that cueing people to reflect on their emotional experiences using their name quickly changes the way that emotions are represented, allowing people to reflect on the self similarly to how they reflect on others. This framework dovetails with the language-as-context view of emotion, which suggests that language rapidly shapes people’s emotional experiences^23^. Critically, these ideas were borne out in the current data, which indicated that third-person self-talk led to reduced activity in two markers of self-referential emotional processing—the LPP and the medial prefrontal cortex^9, 11, 12^. Most important to the aims of this study, however, these patterns of neural activity were observed in the absence of increases in cognitive control activations.

Although the current findings suggest that third-person self-talk does not recruit *cognitive control* relative to first-person self-talk, our findings should not be misconstrued as suggesting that cognitive processing is not involved in third-person self-talk. Indeed, diverse theories of emotion agree that basic cognitive operations are involved in generating emotional experience e.g.^24, 25^. What our findings do suggest is that third-person self-talk does not recruit the network of brain regions typically implicated in the cognitive control of emotion^20^.

It is important to note that we did not observe any modulation of the amygdala as a function of using one’s own name in Study 2. Failure to find this modulation was surprising given prior research that has consistently shown reductions in amygdala activity by reappraisal strategies aimed at regulating negative affect^20^. However, several studies that have used memory-based paradigms for eliciting emotional reactivity have failed to show significant activations and modulation of the amygdala^26, 27^. Thus, it is possible that failure to see modulation of the amygdala in this study was a function of the paradigm employed. In this vein, it is noteworthy that the bulk of the studies included in the meta-analysis we used to identify brain regions that reappraisal strategies modulate consisted of studies using picture-based emotion elicitation paradigms. However, the decrease in LPP by third-person self-talk in Study 1 does point to the possibility that using one’s own name decreases amygdala activity, as the amplitude of the LPP has been consistently linked with amygdala activity^28, 29^. Regardless, together, the current findings provide preliminary evidence pointing to the effectiveness of third-person self-talk for reducing neural markers of self-referential emotional processing. The exact markers that are modulated likely reflect the type of emotional stimulus encountered – in the present case, either externally presented pictured scenes (Study 1) or internally generated memories (Study 2).

Because of its simplicity and effectiveness, third-person self-talk could prove useful for promoting emotion regulation in daily life. Third person self-talk would be easy to disseminate at a large scale and, based on the current findings, should be fairly easy to implement. The ERP and fMRI signatures of third-person self-talk implementation identified here could be important screening metrics to identify individuals who might benefit the most from using this tool (e.g., individuals who are excessively self-focused in the face of negative experiences), and to track the effects of interventions aimed at cultivating it.

## Methods and Materials

### Study 1

#### Participants and stimuli

Thirty-seven undergraduates were run through an emotional picture-viewing task in exchange for partial course credit. Informed consent was obtained from all participants. Eight participants were excluded from analyses because of excessive artifacts due to eye movements resulting in rejection of 100% of trials (n = 3) and body movements resulting in rejection of** > **60% of trials (n = 5). The final sample submitted to analysis included 29 (18 female) participants. The mean age was 18.86 (*SD* = 1.15). All participants were native English speakers. All procedures were performed in accodance with the relevant guidelines and regulations and approved by Michigan State University’s Institutional Review Board.

The stimulus set consisted of 60 neutral and 60 negative images selected from the International Affective Picture System (IAPS). The following images were included: 1050, 1200, 1300, 1525, 1930, 2036, 2102, 2110, 2190, 2200, 2206, 2210, 2214, 2215, 2230, 2320, 2357, 2383, 2393, 2495, 2570, 2661, 2683, 2688, 2692, 2694, 2703, 2710, 2716, 2751, 2753, 2799, 2800, 2810, 2811, 2840, 3001, 3010, 3120, 3181, 3213, 3216, 3220, 3230, 3301, 3350, 3500, 3530, 3550, 5500, 5531, 5971, 6021, 6150, 6211, 6212, 6242, 6300, 6312, 6313, 6315, 6550, 6563, 6821, 6825, 6838, 7000, 7002, 7003, 7004, 7006, 7009, 7010, 7012, 7016, 7018, 7020, 7021, 7025, 7026, 7030, 7031, 7035, 7041, 7050, 7056, 7080, 7100, 7110, 7140, 7150, 7160, 7170, 7175, 7190, 7211, 7217, 7224, 7233, 7235, 7254, 7550, 7620, 7700, 7950, 9250, 9253, 9260, 9410, 9421, 9425, 9428, 9440, 9620, 9622, 9800, 9810, 9903, 9908, 9921.

Normative ratings indicated that negative images were rated as both more negative (Negative: *M* = 2.50, *SD* = 0.73; Neutral: *M* = 4.96, *SD* = 0.41; *t*(118) = 22.64, *p* < .001) and more arousing (Negative: *M* = 6.06, *SD* = 0.74; Neutral: *M* = 3.04, *SD* = 0.68; *t*(118) = 23.22, *p* < .001) than neutral images. Images used for the First and Third person conditions did not differ on either dimension (*ts*(118) < 1, *ps* > .44).

The task was administered on a Pentium D class computer, using E-Prime software (Psychology Software Tools; Pennsylvania, US) to control the presentation and timing of all stimuli. Each picture was displayed in color and occupied the entirety of a 19in (48.26 cm) monitor. Participants were seated approximately 60 cm from the monitor.

#### Procedure

Participants completed a cue-picture paradigm, similar in format to previous research on emotion regulation^9, 16^. The task comprised two blocks, one for each Instruction Type (i.e. First- vs. Third-Person). The order of Instruction Type block was counterbalanced across participants. Each block contained 60 cue-picture trials comprised of 30 neutral and 30 negative IAPS images equally crossed with the two Instruction Type cues. The order of cue-picture trials was random. Fig. 1 illustrates a schematic of the trial structure across both blocks. For each trial, participants first viewed an instruction phrase (“First-Person” or “Third-Person”) for 2 s that directed them how to think about the following picture. “First-Person” indicated that the participant should reflect on their feelings elicited by the pictures using the pronoun “I” as much as possible (i.e., “…ask yourself ‘what am I feeling right now?’”). “Third-Person” indicated that the participant should reflect on their feelings elicited by the pictures using their own name as much as possible (i.e., “…ask yourself ‘what is [participant’s name] feeling right now?”). Participants were further told not to generate unrelated thoughts or images to alter their responses. For all instructions, participants were told to view the pictures for the entire display period and to not look away or close their eyes. After the instruction phrase, a blank screen was presented for 500 ms followed by a centrally presented white fixation cross lasting 500 ms. Following the fixation cross, the IAPS images were displayed for 6 s. A period of 2.5 s was inserted between the offset of images and the presentation of the next instruction phrase during which time participants were instructed to relax and clear their minds.

Participants completed practice trials before each block to familiarize themselves with the timing of events and instructions. The experimental task then included 120 cue-picture trials: 30 Neutral/First-Person, 30 Neutral/Third-Person, 30 Negative/First-Person, and 30 Negative/Third-Person. At the end of each block participants were asked to rate the extent to which they used the first- vs. third-person pronouns when focusing on their feelings using a 1 (not at all) to 7 (all the time) likert scale.

#### Psychophysiological Recording and Data Reduction

Continuous electroencephalographic (EEG) activity was recorded using the ActiveTwo Biosemi system (Biosemi, Amsterdam, The Netherlands). Recordings were taken from 64 Ag-AgCl electrodes embedded in a stretch-lycra cap. Additionally, two electrodes were placed on the left and right mastoids. Electro-oculogram (EOG) activity generated by eye-movements and blinks was recorded at FP1 and three additional electrodes placed inferior to the left pupil and on the left and right outer canthi. During data acquisition, the Common Mode Sense active electrode and Driven Right Leg passive electrode formed the ground per Biosemi design specifications. The function of the CMS-DRL loop, in addition to forming a reference, is simply to constrain the common mode voltage (i.e. the average voltage of the participant), which limits the amount of current that can possibly return to the participant. Bioelectric signals were sampled at 512 Hz.

Initial electrical signal processing was performed offline using BrainVision Analyzer 2 (BrainProducts, Gilching, Germany). Scalp electrode recordings were re-referenced to the mean of the mastoids and band-pass filtered (cutoffs: 0.01–20 Hz; 12 dB/oct rolloff). Ocular artifacts were corrected using the method developed by Gratton and colleagues^30^. Cue- and picture-locked data were segmented into individual epochs beginning 500 ms before stimulus onset and continuing for 3 s and 6 s, respectively. Physiologic artifacts were detected using a computer-based algorithm such that trials in which the following criteria were met were rejected: a voltage step exceeding 50 μV between contiguous sampling points, a voltage difference of 300 μV within a trial, and a maximum voltage difference of less than 0.5 μV within 100 ms intervals. The average activity in the 500 ms window prior to cue and picture onset served as the baseline and was subtracted from each data point subsequent to cue and picture onset.

#### Data Analysis Strategy

For analysis of the LPP, we generated topographically organized clusters of electrodes in order to reduce the spatial dimensions of the dataset. Following the suggestion of Dien and Santuzzi^31^, we computed the following clusters using the average of the noted electrodes: Left-Anterior-Superior (AF3, F1, F3, FC1 and FC3), Right-Anterior-Superior (AF4, F2, F4, FC2 and FC4), Left-Anterior-Inferior (AF7, F5, F7, FC5, and FT7), Right-Anterior-Inferior (AF8, F6, F8, FC6, and FT8), Left-Posterior-Superior (CP1, CP3, P1, P3, and PO3), Right-Posterior-Superior (CP2, CP4, P2, P4, and PO4), Left-Posterior-Inferior (CP5, P5, P7, PO7, and TP7), and Right-Posterior-Inferior (CP6, P6, P8, PO8, and TP8). Because the LPP is a broad and sustained waveform we conducted two analyses of its amplitude across time, following convention^9, 11^. Specifically, research indicates that early time windows (300–1000 ms) index attention allocation whereas later time windows (>1000 ms) index memory and meaning-making stages^11^. As such, we submitted the early LPP amplitude elicited between 400–1000 ms to a 2 (Time: 400–700 and 700–1000 ms) X 2 (Valence: Neutral and Negative) X 2 (Self-talk Strategy: First-Person and Third-Person) X 2 (Hemisphere: Left and Right) X 2 (Anterior and Posterior) X 2 (Superior and Inferior) repeated measures analysis of variance (rANOVA). This allowed us to capture the early, attention-related LPP before the sustained portion of the LPP begins around 1000 ms [see also refs 9 and 18]. To understand the timing of memory and meaning-making processes, we submitted the sustained portion of the LPP to a 5 (Time: 1–2 s, 2–3 s, 3–4 s, 4–5 s, and 5–6 s) X 2 (Valence: Neutral and Negative) X 2 (Self-Talk Strategy: First-Person and Third-Person) X 2 (Hemisphere: Left and Right) X 2 (Anterior and Posterior) X 2 (Superior and Inferior) rANOVA [see also refs 9 and 18].

The SPN was identified and quantified at fronto-central electrodes (F1, Fz, F2, FC1, FCz, FC2) as in previous work^9, 14^. Previous work indicates that early enhancement of the SPN reflects orienting to the preceding cue whereas later increases reflect anticipation of and preparation to act on the upcoming imperative stimulus^14^. Therefore, consistent with past research^9, 14^, the early SPN was defined as the average voltage in the 300–2300 ms time window post-cue onset and the late SPN was defined as the average voltage in the 2300–3000 ms time window post-cue onset, the latter corresponding to the 700 ms immediately preceding picture onset during which time participants viewed the blank screen and fixation cross separating the cue and the picture. The early and late SPN were submitted to separate 2 (Valence: Neutral and Negative) X 2 (Self-talk Strategy: First-Person and Third-Person) rANOVAs.

Additional analyses of the LPP and SPN effects are available in the supplemental materials.

### Study 2

#### Participants

Fifty-two individuals (32 females) were recruited for participation. Participants were recruited via flyers posted and advertisements posted on Facebook and Craig’s List. The sample consisted of 71.15% Caucasian, 15.38% Asian, 7.69% African American, 1.92% Native American, and 3.85% other. The mean age was 20.19 (*SD* = 2.66). All participants were right-handed native English language speakers and received up to $50 for their participation. Informed consent was obtained from all participants. All procedures were performed in accordance with the relevant guidelines and regulations and approved by the University of Michigan’s Institutional Review Board.

Data from two participants was excluded from all analyses due to a technical malfunction during the fMRI task. Specifically, one participant did not see their name appear during the fMRI task, and the scan data from one participant was not properly saved. Thus, all of the descriptive data we report from here on pertains to the subsample of fifty participants that were included in our analyses.

#### Screening Session: Stimuli Harvesting

Similar to prior studies that have used script-driven methods, cue phrases were used to trigger the recall of negative autobiographical memories in the scanner. Following protocols implemented in prior research^32, 33^, we obtained memory cues by asking participants to recall and then describe in writing eight highly arousing negative autobiographical experiences during a screening held before the scan session (*M*_*days*_ = 10.81; *SD*_*days*_ = 7.87). To qualify for the study, participants had to have experienced eight unique negative events which led them to feel intensely distressed each time they thought about them. To ensure that this criterion was met, a memory was considered “eligible” if participants rated it above the midpoint of a 1 (not intense) to 9 (extremely intense) arousal scale (*M* = 7.49, *SD* = 1.07) and below the midpoint on a 1 (extremely negative) to 9 (extremely positive) valence scale (*M* = 2.05; *SD* = 0.97).

#### fMRI Negative Emotion Elicitation Task

The Negative Emotion Elicitation task was modeled after prior fMRI and behavioral research indicating that cueing people to recall autobiographical negative experiences is an effective way of reactivating intense idiosyncratic negative emotion e.g.^32, 33^. The stimuli for this task consisted of cue phrases (e.g., rejected by Marc; party with Ted) that appeared in the center of each screen, which directed participants to focus on a specific negative past experiences. Participants generated these cue-phrases on their own, before the day of scanning, using a procedure developed in prior research^32, 33^. Specifically, they first wrote about each of their experiences. Subsequently, they were asked to create a cue-phrase that captured the gist of their experience (e.g., barking dog). They were reminded of the cues they generated and the experiences they referred to on the day of scanning following established procedures.

#### Task Training

Before scanning, participants were told that each trial would begin with a fixation cross, which they were asked to stare directly at. Next, they were told that they would see a linguistic cue, which would instruct them how to introspect (i.e., using *I* or *their* name) during the trial. Next, they were told that they would see another fixation-cross followed by a memory cue-phrase that they generated during the previous session. When they saw the memory cue they were asked to reflect over that memory using the part of speech (I or their own name) that was previously presented to them. To ensure that they used the correct part of speech when introspecting about each memory the same linguistic cue that was presented earlier during the trial appeared beneath the memory cue on the bottom of the screen. Subsequently, participants were told they would have 3-s to rate how they felt using a five-point scale (1 = not at all negative; 5 = very negative; see Fig. 2 for an illustration of the task).

#### Functional MRI Acquisition and Analysis

Whole-brain functional data were acquired on a GE Signa 3-Tesla scanner. A spiral sequence with 40 contiguous slices with 3.44 × 3.44 × 3 mm voxels (repetition time (TR) = 2000 ms; echo time (TE) = 30; flip angle = 90°; field of view (FOV) = 22 cm) was used to acquire functional T2* weighted images. Structural data were acquired with a T1-weighted gradient echo anatomical overlay acquired using the same FOV and slices (TR = 250 ms, TE = 5.7 ms, flip angle = 90°). We also collected a 124-slice high-resolution T1-weighted anatomical image using spoiled-gradient-recalled acquisition (SPGR) in steady-state imaging (TR = 9 ms, TE = 1.8 ms, flip angle = 15°, FOV = 25**–**26 cm, slice thickness = 1.2 mm).

Functional images were corrected for differences in slice timing using 4-point sinc-interpolation^34^ and were corrected for head movement using MCFLIRT^35^. Each SPGR anatomical image was corrected for signal in-homogeneity and skull-stripped using FSL’s Brain Extraction Tool^36^. These images were then segmented with SPM8 (Wellcome Department of Cognitive Neurology, London) into gray matter, white matter and cerebrospinal fluid and normalization parameters for warping into MNI space were recorded. These normalization parameters were applied to the functional images maintaining their original 3.44 × 3.44 × 3 mm

Functional scans were physio-corrected using retroicorr resolution^37^. The functional scans were further preprocessed with SPM8’s, slice-time correction, and realignment processing functions. The segmented normalization parameters were then applied to the functional scans to warp them into MNI space. Finally, the functional images were spatially smoothed using a 8-mm full-width at half-maximum Gaussian kernel.

Statistical analyses were conducted using the general linear model framework implemented in SPM8. Boxcar regressors, convolved with the canonical hemodynamic response function, modeled periods for the 2-s linguistic cue (“I” or their own name), 4-s fixation cross, 15-s memory cue, and 3-s affect rating. The fixation-cross epoch was used as an implicit baseline.

#### Whole Brain Analysis

Voxelwise statistical parametric maps summarizing differences between trial types were calculated for each participant and then entered into random-effects group analyses, with statistical maps thresholded at *P* < 0.05 FWER-corrected for multiple comparisons across gray and white matter. This correction entailed a primary threshold of *P* < 0.005, with an extent threshold of 146 voxels, which was determined using a Monte Carlo simulation method and was calculated using 3DClustSim^38^. This technique controls for the FWER by simulating null datasets with the same spatial autocorrelation found in the residual images and creates a frequency distribution of different cluster sizes. Clusters larger than the minimum size corresponding to the a priori chosen FWER are then retained for additional analysis. This cluster-based method of thresholding is often more sensitive to activation when one can reasonably expect multiple contiguous activated voxels^38, 39^, and is widely used in fMRI research. Our principle analyses contrasted the hemodynamic response on trials on which participants recalled negative autobiographical experiences and then analyzed their feelings surrounding the events using ***I*** or ***their own name***.

#### Region of Interests (ROIs)

We performed region of interest analyses on three sets of brain regions: (a) brain regions that support self-referential processing (e.g., medial prefrontal cortex) (b) brain regions involved in emotional reactivity (e.g., the amygdala), and (c) brain regions that support the effortful, cognitive control of emotion (e.g., posterior dorsomedial prefrontal cortex and bilateral dorsolateral PFC and ventrolateral PFC, and posterior parietal cortex).

The coordinates for each set of ROIs were derived from meta-analyses. Specifically, the self-referential processing ROIs were obtained from a meta-analysis based on data from 28 neuroimaging studies that contrasted the evaluation of the self (i.e., thinking about one’s own traits) versus close and distant others (i.e., thinking about others’ traits^18^). To conservatively select the ROIs of interest for this analysis, only regions that were active for self vs. *both* close and distant others were included, as the subject’s own name does not neatly fit into either category of the other. The ROIs corresponding to brain regions involved in emotional reactivity and support the implementation of cognitive emotion regulation were obtained from a recent meta-analysis based on data from 48 neuroimaging studies of reappraisal, the most commonly studied form of cognitive emotion regulation^20^. We used Robert Welsh’s simpleroibuilder to create spherical ROIs around the peak voxels from prior work. The spherical ROIs were generated to have the same volume as those from the original studies. All ROIs were small volume corrected to an FWER equivalent to p < .05.

## Electronic supplementary material

Supplementary Information
