# Supplementary material for: Third-person self-talk facilitates emotion regulation without engaging cognitive control: Converging evidence from ERP and fMRI
Source: Sci Rep. 2017 Jul 3;7:4519. doi: 10.1038/s41598-017-04047-3 (PMC5495792; doi:10.1038/s41598-017-04047-3)
Supplement: Supplementary file 1 — Supplementary Information [file 41598_2017_4047_MOESM1_ESM.pdf]

SUPPLEMENTARY INFORMATION FOR

Third-person self-talk facilitates emotional control without engaging cognitive control: Converging evidence from ERP and fMRI

Jason S. Moser[1*], Adrienne Dougherty[2], Whitney I. Mattson[2], Benjamin Katz[2], Tim P. Moran[1,5], Darwin Guevarra[2], Holly Shablack[2], Ozlem Ayduk[3], John Jonides[2], Marc G. Berman[4], Ethan Kross[2*]

[1]Department of Psychology, Michigan State University, East Lansing, Michigan.

[2]Department of Psychology, University of Michigan, Ann Arbor, Michigan.

[3]Department of Psychology, University of California, Berkeley, California

[4]Department of Psychology, University of Chicago, Chicago, Illinois

[5]School of Medicine, Emory University, Atlanta, Georgia

*Correspondence to: jmoser@msu.edu or ekross@umich.edu

Supplemental Results for Study 1

Figure S1 displays the morphology and topography of the LPP with respect to its modulation by Time and Valence.

LPP 400-700 and 700-1000

There was a significant effect of Time (F (1,28) = 26.33, $p < .001$, $\eta^2_p = .49$) such that the LPP grew larger over time. There was also a significant effect of Anterior/Posterior (F (1,28) = 87.78, $p < .001$, $\eta^2_p = .76$) such that the LPP was larger at posterior sites. The significant Time X Anterior/Posterior effect (F (1,28) = 34.35, $p < .001$, $\eta^2_p = .55$) indicated that the LPP was larger at posterior sites later in the time window. The Time X Superior/Inferior effect (F (1,28) = 17.86, $p < .001$, $\eta^2_p = .39$) indicated that the LPP was larger at superior locations later in time. The Anterior/Posterior X Superior/Inferior interaction (F (1,28) = 7.56, $p = .01$, $\eta^2_p = .21$) indicated that the LPP was larger at superior than inferior sites especially in anterior regions. The Time X Anterior/Posterior X Superior/Inferior interaction (F (1,28) = 10.77, $p < .01$, $\eta^2_p =$ .28) showed that the LPP was larger at superior sites in the anterior region at both time windows, but was relatively larger at superior sites later in the time window at posterior sites.

The Time X Laterality effect (F (1,28) = 9.82, $p < .01$, $\eta^2_p = .26$) indicated that the LPP was larger at right locations earlier but larger at left locations later in time. The Time X Superior/Inferior X Laterality interaction (F (1,28) = 10.84, $p < .01$, $\eta^2_p = .28$) showed that the LPP was larger at right locations early at inferior sites and no difference between superior sites, but later the LPP was larger at left locations at superior sites more so than at inferior sites. The Laterality X Self-Talk Strategy (F (1,28) = 5.68, $p < .05$, $\eta^2_p = .17$) effect indicated that the LPP was larger at left than right sites for first-person blocks but that the LPP was larger at right than left locations in third-person blocks; neither simple effect was significant, however ($Fs < 1$).

The Time X Valence effect (F (1,28) = 7.66, p = .01, η^2_p = .22) indicated that the valence effect (negative - neutral) was larger at the later time window. The Anterior/Posterior X valence interaction (F (1,28) = 8.78, p < .01, η^2_p = .24) showed that the valence effect (negative - neutral) was larger at posterior sites. The Time X Anterior/Posterior X Valence interaction (F (1,28) = 10.07, p < .01, η^2_p = .27) showed that the valence effect (negative - neutral) was larger at posterior sites especially at the later time window. The Superior/Inferior X Valence effect (F (1,28) = 13.71, p = .001, η^2_p = .33) indicated that the valence effect (negative - neutral) was larger at superior than inferior sites. The Time X Superior/Inferior X valence effect (F (1,28) = 12.77, p = .001, η^2_p = .31) showed that the valence effect (negative - neutral) was larger at superior sites especially at the later time window. Finally, the time X Anterior/Posterior X Superior/Inferior X Laterality X Valence interaction (F (1,28) = 6.82, p < .05, η^2_p = .20) was significant.

Together, the results indicated the typical superior-posterior distribution of the LPP, especially with respect to its modulation by valence. As indicated in the main text, however, there was no significant effect of Self-Talk Strategy (F(1,28) < 1, p = .36, η^2_p = .03) in the early LPP time window.

LPP 1-6s

The main effect of Superior/Inferior (F (1,28) = 12.14, p < .01, η^2_p = .30) was significant such that the LPP was larger at superior sites. The Time X Anterior/Posterior interaction (F (1,28) = 9.10, p < .001, η^2_p = .25) showed that the LPP was larger at posterior sites early and anterior sites later. The Time X Anterior/Posterior X Valence interaction (F (1,28) = 4.60, p < .01, η^2_p = .14) revealed that the valence effect (negative - neutral) begins more posterior and then becomes distributed and more anterior over time, consistent with past results[6]. The

Superior/Inferior X Valence interaction (F (1,28) = 11.69, $p < .01$, $\eta^2_p = .30$) indicated that the valence effect (negative - neutral) was more superior. The Laterality X Valence interaction (F (1,28) = 5.93, $p < .05$, $\eta^2_p = .18$) indicated that there was a larger valence effect (negative - neutral) at left locations. The Time X Laterality X Valence interaction (F (1,28) = 5.35, $p < .01$, $\eta^2_p = .16$) showed that the valence effect (negative - neutral) becomes more left lateralized over time. Critically, as reported in the main text, the Valence x Self-Talk Strategy interaction was significant (F(1, 28) = 5.18, $p = .03$, $\eta^2_p = .16$). Moreover, this interaction was not further modified by location and thus was evenly distributed across the scalp (as this 2-way interaction was not qualified by any higher order interactions involving laterality, caudality, or dorsal/ventral; *Fs* < 2.5, *ps* > .12; see Figure S2 for a visual depiction of the broad scalp distribution of this interaction).

SPN

As indicated in the main text, and consistent with our prediction, results revealed no main effect of Self-Talk Strategy. Figure S3 depicts the scalp distribution of this null effect.

Brain-Behavior Relationships

Finally, we evaluated the relationship between LPP modulation by Type of Self Talk Instruction and participants' ratings of compliance with the self-talk instructions.

Our analysis showed that the degree to which participants showed a larger LPP emotion effect (negative-neutral) in the First-Person block than in the Third-Person block was associated with reports of greater use of the appropriate pronouns across both blocks ($r = .44$, $p < .05$). That is, the expected blunting of the emotion effect in the Third-Person (compared to the First-Person) block scaled with the degree to which participants effectively employed the different personal pronouns in each block.

Supplemental Results for Study 2

Brain Behavior Correlations

We examined the relationship between negative affect and reduced activation in brain areas associated with self-referential processing by (a) extracting average activation for each participant from the functional region of interest from meta-analytic findings (Araujo, Kaplan, & Damasio, 2013), and then (b) correlating this with the difference between each subject's self-reported ratings of emotion in the I trials vs Name trials.

As noted in the text (see Footnote X), initial analyses of the distribution of these difference scores revealed two extreme values corresponding to the I > Name extracted beta values (i.e., scores that were greater than two standard deviations than the sample mean). Subsequent analyses indicated that these two values exerted a relatively large amount of influence on the linear regression, *Cook's Distance* = 0.28, 0.26. Thus, we report the analyses below both with and without these values included, consistent with the reports we result in the main text.

Analyses involved all participants indicated that the correlation between the activation extracted from the left medial prefrontal cortex/anterior cingulate cortex correlated negatively with the drop in self-report negative affect that participants reported experiencing on Name trials compared to I trials (i.e., $I_{\text{distress scores}}$ - $\text{Name}_{\text{distress scores}}$) for both the functional ROI, $R^2 = .08$, $B = -1.25$, $SE = .63$, $t(48) = -2.00$, $p = .05$, and the AAL ROI from whole-brain analysis, $R^2 = .08$, $B = -1.26$, $SE = .60$, $t(48) = -2.10$, $p < .05$. However, subsequent analyses that did not include the two extreme values above revealed no significant brain-behavior correlation for either the functional ROI, $R^2 = .03$, $B = -0.56$, $SE = .47$, $t(48) = -1.18$, $p = .24$, or the AAL ROI, $R^2 = .04$, $B = -0.67$, $SE = .49$, $t(48) = -1.35$, $p = .18$, suggesting that the aforementioned results was not robust.

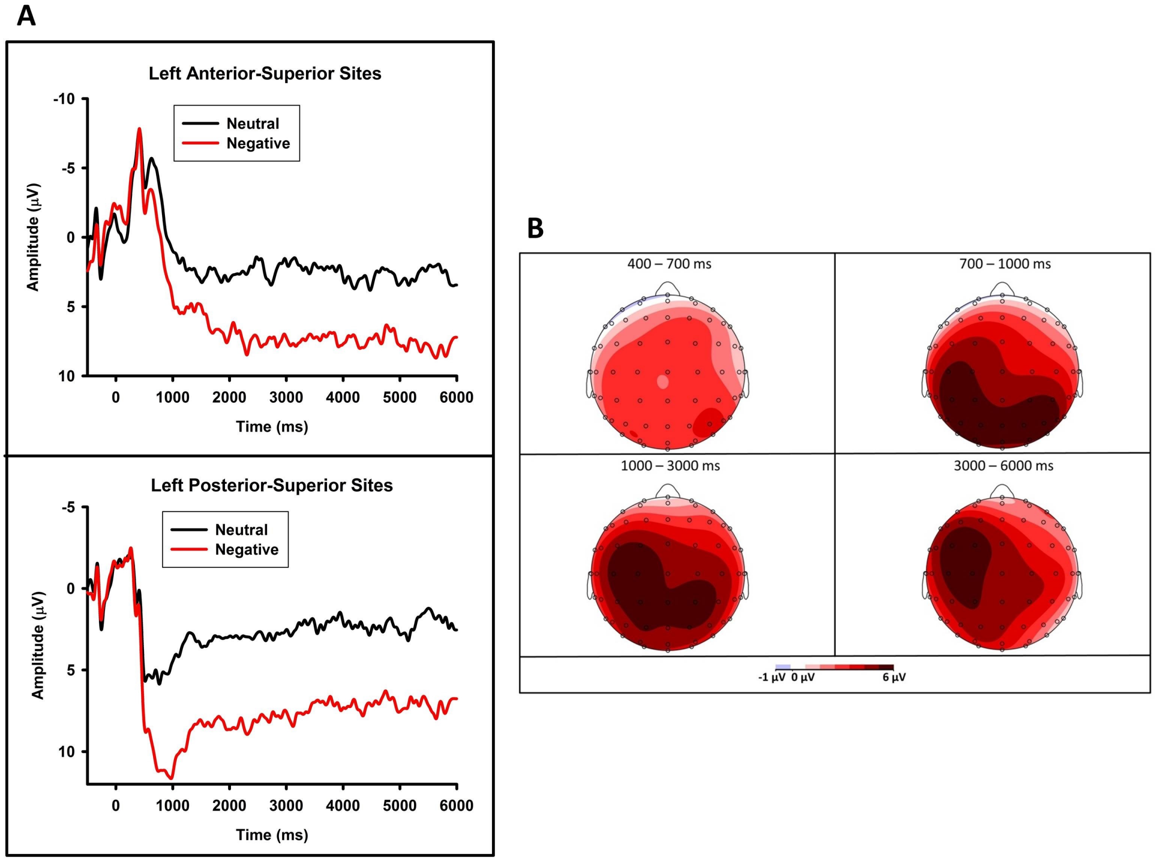

Figure S1. Supplemental ERP Results. (A) Picture-locked ERP waveforms at representative left anterior- and posterior- superior sites depicting the LPP elicited by neutral and negative pictures. Picture onset occurs at 0ms. **(B)** Voltage maps depicting the topographical distribution of the LPP emotion effect (negative – neutral) across time. Darker shades of red indicate a larger emotion effect where negative pictures elicit a larger LPP than neutral pictures.

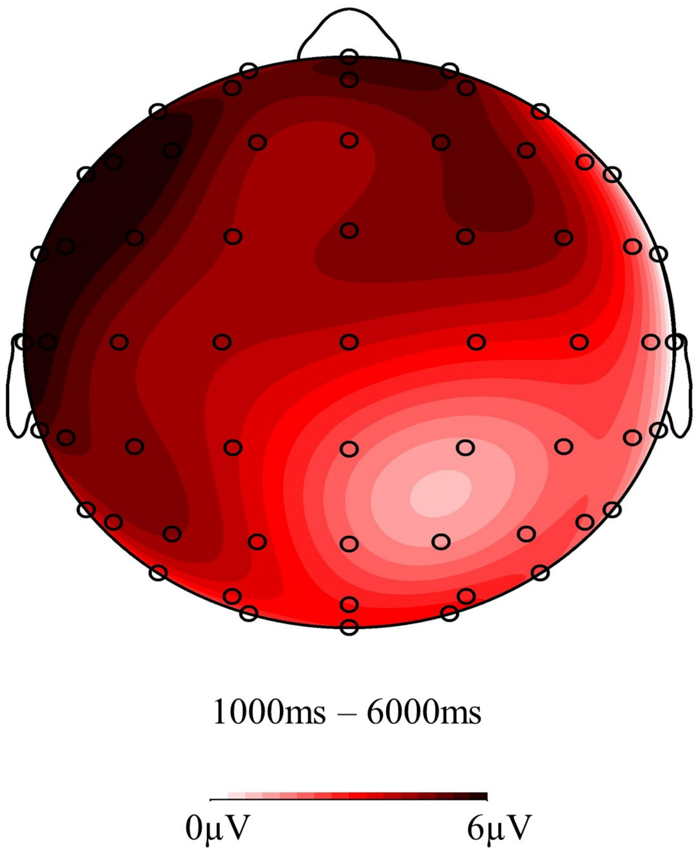

Figure S2. Supplemental ERP Results. Voltage map depicting the broad topographical distribution of the larger negative – neutral LPP difference score for the First-Person compared to Third-Person condition during the late LPP time window (darker shades of red indicate a larger effect).

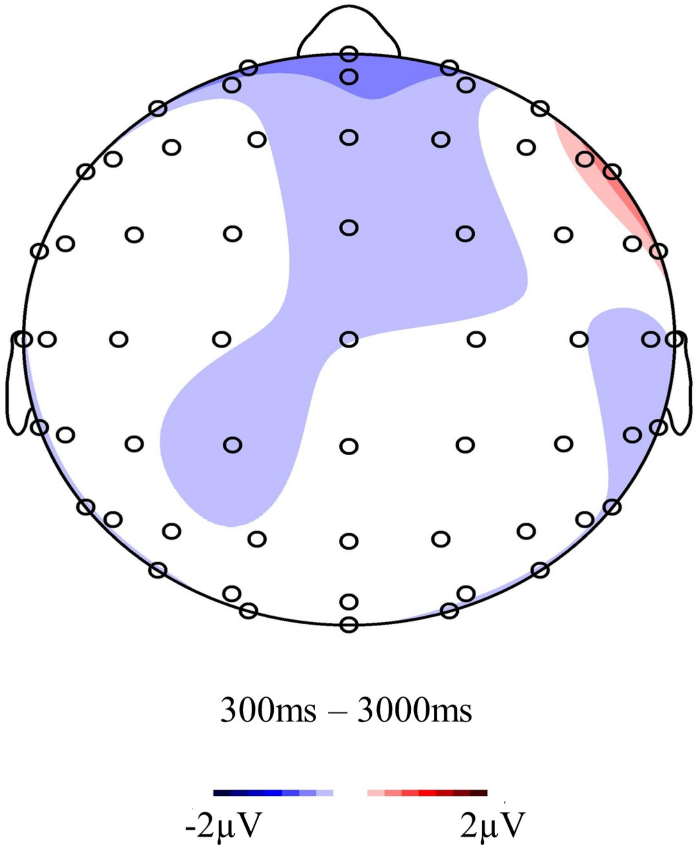

Figure S3. Supplemental ERP Results. Voltage map depicting the topographical distribution of the null main effect of Self-Talk Strategy, averaged across Valence, on the SPN across early and late time windows (300-3000ms total). Darker shades of blue would indicate a larger SPN for Third-Person compared to First-Person Self-Talk, but only small, non-significant differences were observed (shown in light blue).

Supplemental Results for Study 2

Table S1. Study 2 (fMRI) ROI Activation Results

Functional Region of Interest I > Name (sphere radius)	Peak *t (k)*	*p*-value	ROI Center MNI Coordinates x	y	z
Left Medial Preferontal Cortex/ Anterior Cingulate Cortex (13 mm)	4.54 (143)	$p < .0001$	-6	46	20
Left Superior Frontal Gyrus/Middle Frontal Gyrus[†] (7 mm)	N/A	N/A	-22	52	30
Right Middle Frontal Gyrus[†] (6 mm)	N/A	N/A	28	52	26
Left Posteromedial Cortex[†] (5 mm)	N/A	N/A	-4	-50	46
Right Amygdala[†] (9 mm)	N/A	N/A	30	-3	-15
Left Amygdala[†] (9 mm)	N/A	N/A	-18	-3	-15
**Functional Region of Interest (Name > I)**	**Peak (*t, k*)**	***p*-value**	**ROI Center MNI Coordinates x**	**y**	**z**
Middle Frontal Gyrus[†] (10 mm)	N/A	N/A	60	24	3
Inferior Frontal Gyrus[†] (9 mm)	N/A	N/A	51	15	48
Medial Frontal Gyrus[†] (13 mm)	N/A	N/A	9	30	39
Middle Frontal Gyrus[†] (15 mm)	N/A	N/A	-33	3	54
Superior Parietal Lobule[†] (8 mm)	N/A	N/A	63	-51	39
Superior Parietal Lobule[†] (9 mm)	N/A	N/A	-42	-66	42
Middle Temporal Gyrus[†] (9 mm)	N/A	N/A	-51	-39	3
**Whole Brain Clusters of Activation**	**Peak (*t, k*)**	***p*-value**	**Peak MNI Coordinates x**	**y**	**z**

I > Name					
Peak	4.54 (258)	$p < .001$	2	53	22
Sub-peak 1	4.19 (-)	$p < .001$	-5	59	1
Sub-peak 2	3.41 (-)	$p = .001$	19	50	-2
Name > I†	N/A	N/A	N/A	N/A	N/A

Note: † - Region of Interest did not contain voxels above signal or cluster threshold, * - ROI defined by AAL atlas definitions
